# Idiopathic sudden sensorineural hearing loss: evolution in the presence of hypertension, diabetes mellitus and dyslipidemias

**DOI:** 10.1590/S1808-86942010000300015

**Published:** 2015-10-20

**Authors:** Jayson Nagaoka, Marcelo Ferreira dos Anjos, Thales Takeo Takata, Renan Moukbel Chaim, Flavia Barros, Norma de Oliveira Penido

**Affiliations:** 1 MSc – ENT and Head and Neck Surgery Graduate Program – UNIFESP; 2 Fellow – ENT and Head and Neck Department – UNIFESP; 3 Ophthalmologist; 4 MD; 5 PhD. Speech and Hearing Therapist; 6 PhD, Graduate Program Advisor in ENT – UNIFESP-EPM. ENT Department – UNIFESP-EPM

**Keywords:** hypertension, diabetes mellitus, sensorineural, hearing loss, sudden hearing loss

## Abstract

Retrospective study aiming at evaluating the interference of associate diseases in the evolution and prognosis of idiopathic sudden sensorineural hearing loss.

**Materials and methods:** Case-Control Study. Thirty-five patients with idiopathic sudden sensorineural hearing loss were divided in two groups, one of them with associate diseases (hypertension, diabetes mellitus and dyslipidemias), and another one without co-occurrence of such diseases. The groups were evaluated regarding: age, gender, associate diseases, presence of tinnitus, dizziness and ear fullness sensation, presence of cerebral microangiopathy observed in magnetic resonance imaging, ophthalmoscopic findings, treatment onset, improvements in audiometric findings and at speech discrimination tests. Statistical analysis of data was performed.

**Results:** The associate disease group showed higher ages, cerebral microangiopathy observed in magnetic resonance imaging and later improvement in speech discrimination tests, being this difference statistically significant.

**Conclusion:** Idiopathic sudden sensorineural hearing loss co-occurring with hypertension, diabetes mellitus or dyslipidemias, in older patients, is associated with a higher prevalence of cerebral microangiopathy, revealed by magnetic resonance imaging, and associated with a slower hearing recovering, showed by later improvements in speech discrimination tests.

## INTRODUCTION

Hearing loss is one of the most common complaints in ENT offices today; however, it is still challenging. The evaluation of patients with proven hearing loss, especially when the loss is sudden, must be detailed, trying to identify possible associated causes and symptoms, as well as treatment, which should be instated as soon as possible.

The name sudden hearing loss is already established, but unfortunately it is a very inaccurate and vague term, often times being interpreted as a nosological entity instead of simply a symptom. That is the reason why we prefer to use the term sudden sensorineural hearing loss.

We define sudden sensorineural hearing loss as being a sensorineural hearing loss above 30dB in at least three adjacent frequencies and of sudden onset or in up to 72 hours^1^.

Etiology, as well as any other symptoms must be investigated systematically, since many diseases may be involved. Neoplasias, vascular alterations, immunemediated disorders, Meniere's disease, membrane rupture and infectious diseases – viral or bacterial in origin, seem to be implicated^2^. Nonetheless, often times, the etiological diagnosis is not possible even with the tests available today. In this case, the sudden sensorineural hearing loss is classified as idiopathic.

The role of viral infections has been challenged. Studies have found cochlear alterations in temporal bones of patients with sudden hearing loss, similar to the ones associated with viral infections or even a larger presence of virus in the nasopharyngeal material in patients with sudden hearing loss^3^,^4^. These findings would suggest viral etiology for the sudden hearing loss, nonetheless, so far, we have been unable to confirm the clinical diagnosis in these findings, since it would be necessary to have histopathology studies of the temporal bone – which is not feasible in vivo, and the search for virus in the inner ear – which is not yet systematized. Concurrently, other authors found evidence that the viral infection would not be the cause of the sudden hearing loss^5^.

In the treatment approach of the patients, although with very little support in the literature, steroids are most used. In their study, Wilson6 noticed a significant improvement in those patients who used steroids instead of placebo, and such evidence has not been confirmed through a systematic review of the literature^7^. Anyway, treatment must be instated as soon as possible, with the best results being seen in up to seven days^8^.

In the assessment of patients with sensorineural hearing loss, systemic disorders, especially systemic hypertension, diabetes mellitus and dyslipidemias are directly or indirectly implicated, since there is no consensus of this association regarding sudden sensorineural hearing loss.

Penido et al.^1^ observed during follow up of their patients with sudden sensorineural hearing loss that the worst results in treatment happened to older patients and those who had associated disorders: systemic arterial hypertension, diabetes mellitus and dyslipidemias. However it did not differentiate whether these associated diseases and age were factors which had an isolated or joint impact in the evolution of these patients.

The assessment of the role these associated diseases play is very important in order to have a better understanding regarding prognosis as well as a program for a better treatment strategy regarding the patients with sudden idiopathic sensorineural hearing loss.

## METHOD

We did a case-controlled study with the patients being followed in the sudden hearing loss ward between 2000 and 2007.

The project was evaluated and approved by the Ethics in Research Committee of the Institution, registered under No. 1871/06.

Of the 177 patients diagnosed with sudden sensorineural hearing loss, 95 were males and 82 females. After the investigation, 13 patients (7.35%) had a defined etiological diagnosis. The remaining 164 patients (92.65%) were classified as having sudden idiopathic sensorineural hearing loss.

Of these 164 patients, we selected 35 individuals (21.34%) to participate in the study. The other patients did not fit the inclusion criteria and were, therefore, taken off the study.

The inclusion criteria we adopted were:

– idiopathic sensorineural hearing loss of sudden onset, or installed in up to 72 hours, seen at the otology department before 30 days after symptom onset.
– follow up at the sudden hearing loss ward for at least six months.

The exclusion criteria we used were:

– Etiological study of the established hearing loss.
– Use of prior treatment.
– Systemic disorders other than systemic arterial hypertension, diabetes mellitus and dyslipidemias, already known or identified during follow up.
– Follow up interruption.
  For evaluation purposes, the selected patients were broken down in two groups: group with associated disease and group without associated disease.
– Group with associated disease: made up of patients with sudden idiopathic sensorineural hearing loss associated with systemic arterial hypertension, diabetes mellitus and dyslipidemias, alone or associated.
– Group without associated disease: made up of patients with sudden idiopathic sensorineural hearing loss without the presence of associated diseases.

The patients seen in the hearing loss ward went through a general medical interview and complete physical exam. In this interview, the patients were asked about symptoms such as tinnitus, dizziness and full ear, as well as hearing loss duration.

The treatment used in all the patients was made up of prednisone in the dose of 1mg/kg/day, for at least seven days, with a progressive dose reduction, and pentoxifylline in the dose of 1,200mg divided in three doses, maintained for at least eight weeks.

The patients were submitted to magnetic resonance tests in their right ears, using gadolinium as a contrast medium, and blood tests: CBC, fasting glycemia, creatinine, total cholesterol and fractions, triglycerides, dosage of free T4.

The hearing assessment of the patients was carried out by a MAICO MA-41 audiometer and all the tests were carried out by the same speech and hearing therapist. We also assessed initial audiometric patterns, after one month of treatment and that again six months after treatment or later. The auditory evaluation was made up of tonal audiometry, vocal audiometry with speech recognition index (SRI) and immittance measures with stapes reflexes study.

The fundus of the eye test was always carried out by the same ophthalmologist. The alterations were classified according to the ETDRS (Early Treatment Diabetic Retinopathy Study) classification.

In the anamneses of the diabetic patients with arterial hypertension or dyslipidemias, the patients were also investigated about the duration of the disease and also treatment instated. Also regarding the diabetic patients, we carried out a tactile and thermal sensitivity evaluation, studying the presence of peripheral neuropathy.

The auditory recovery assessment was carried out by means of an improvement rate, which measures the auditory gain in percentage and uses the contralateral side as reference. Calculated according to the following formula:

Improvement rate (%) = (initial threshold – final threshold × 100 /initial threshold – Contralateral ear threshold)

The auditory thresholds were calculated using the mean values of frequencies 50, 500, 1000, 2000, 4000, 6000 and 8000 Hz.

The threshold was obtained based on the last audiometry held in at least six months after treatment onset, and in all the cases the auditory thresholds of the contralateral ear were below 30dB.

The variables analyzed in our study were: age, gender, associated disease, tinnitus, dizziness and ear fullness, the presence of cerebral microangiopathy seen at the MRI, alterations in the fundus of the eye exam, treatment onset, hearing improvement rate, initial speech recognition index (SRI), after fifteen days, one month and six months or more of follow up.

The results obtained were taken to statistical analysis. In order to compare the two groups studied regarding the categorical variables belonging to the study, we used the Fisher's exact test. Regarding variables age, improvement rate and treatment onset we used the t Student test for unrelated samples; and in order to study the behavior of the SRI variable in time for each group, we used the variance analysis model with repeated measures.

In all the analysis, the significance level was 5% (p<0.05).

## RESULTS

The analysis was initially made up of a general discussion of the information collected. The gender distribution in the two groups was similar. The group with associated disease was made up by ten men and ten women and the group without associated disease by seven men and eight women.

Table 1 depicts the mean values of age, improvement rate and time of treatment onset of the sudden sensorineural hearing loss in the two groups, with their respective standard deviation and maximum and minimum values.

**Table 1:** Descriptive measures of the variables: Age, improvement rate and treatment onset, according to each group.

Group		Age	Improvement rate (%)	Treatment onset time
	Mean	60,35	36,31	11,10
With associated	SD	9,93	25,72	8,79
disease	Minimum	43,00	1,47	1,00
	Maximum	76,00	100,00	30,00
	Mean	41,60	40,58	14,60
Without	SD	16,82	29,20	11,27
associated				
disease	Minimum	22,00	-4,60	2,00
	Maximum	72,00	93,33	30,00
	Mean	52,31	38,14	12,60
	SD	16,13	26,93	9,93
Total				
	Minimum	22,00	-4,60	1,00
	Maximum	76,00	100,00	30,00

Table 2 depicts the mean values of the speech recognition index (SRI) throughout time, with their respective standard deviation and maximum and minimum values.

**Table 2:** SRI variable values along time, according to each group and in relation to the total number of patients assessed in the study.

Group		Onset	15days	30days	180 days or more
	Mean	21,60	34,32	40,00	44,42
With associated	SD	27,33	40,47	40,90	39,71
disease	Minimum	0,00	0,00	0,00	0,00
	Maximum	80,00	92,00	96,00	100,00
	Mean	17,60	44,62	53,87	59,73
Without associated disease	SD	28,07	43,48	38,12	39,84
	Minimum	0,00	0,00	0,00	0,00
	Maximum	84,00	100,00	100,00	100,00
	Mean	19,89	38,50	45,94	51,18
Total	SD	27,31	41,34	39,77	39,91
	Minimum	0,00	0,00	0,00	0,00
	Maximum	84,00	100,00	100,00	100,00

The fundus of the eye exam only proved altered in four patients of the sample and they all belonged to the group with associated disease. Three patients were hypertensive and diabetic, and one was only diabetic, in all the patients the alterations were mild diabetic retinopathies, characterized by the presence of retina microaneurysms. Table 3 depicts MRI alterations found for both groups.

**Table 3:** Sample distribution according to the MRI variable per group.

Group			
MRI	With associated disease	Without associated disease	Total
Normal	9	13	22
Microangiopathy	11	2	13
Total	20	15	35

In the group with associated disease, 12 patients were hypertensive, two diabetics and three with dyslipidemias. In three patients there was association between arterial hypertension and diabetes mellitus.

In the evaluation of the patients, 83% had tinnitus, 51% had dizziness and 20% of the patients in our sample had ear fullness. And in the subsequent follow up of the patients we identified the recurrence of the sudden idiopathic sensorineural hearing loss in two patients from the group of patients with associated disease, the recurrence happened after six months of follow up.

**Figure 1:**
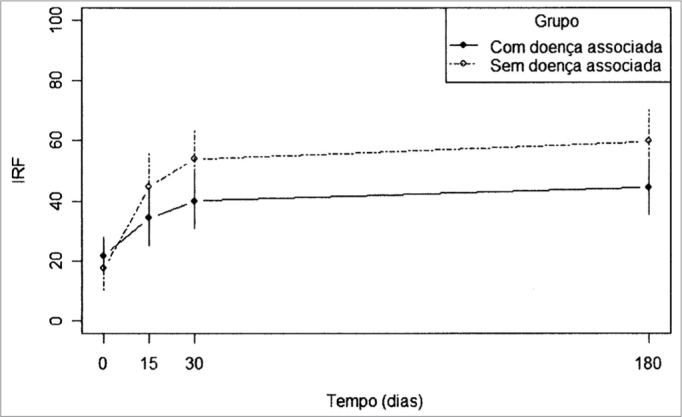
. Mean values of the SRI variable for each study group.

In Fig.1 we see the mean values of speech recognition index (SRI) in the two groups along time.

In order to compare the two groups studied in relation to the categorical variables belonging to the study we used the Fisher's exact test. The results depicted on Table 4 let us state that the groups “with associated disease” and “without associated disease” differ as to the result from the magnetic resonance imaging study.

**Table 4:** Results from the comparison of the groups according to the categorical variables of the study.

Variable	Descriptive level
Gender	0.558
Eye fundus	0.159
MRI	0.013*
Dizziness	0.111
Tinnitus	0.481
Ear fullness	0.101

* p< 0,05

In order to compare the two groups studied in relation to variables Age, Improvement rate and Treatment onset time we used the t Student for non-related samples. The results depicted on Table 5 let us state that groups with associated disease” and “without associated disease” differ as to the mean age.

**Table 5:** Results from the comparison between the two groups in relation to the numerical variables of the study.

Variable	Descriptive level
Age	0.001*
Improvement rate (%)	0.650
Treatment onset time	0.309

* p ≤ 0,05

In order to study the behavior of the speech recognition index (SRI) variable throughout time, in each group, we used the variance analysis model with repeated measures. The results shown on Tables 6 and 7 let us state that: For the group “with associated diseases”, the mean values obtained from 30 and 180 days are equal, but different from the mean value found in the beginning of the study.

**Table 6:** Results from the application of the variance analysis model with repetitive measures.

Effect	Descriptive level
Group	0.789
Time	0.001*
Interaction	0.022*

* p ≤ 0,05

**Table 7:** Results from the comparison between the measures obtained from the SRI at different instances, according to each group.

Group	Times compared	Descriptive level
With disease	Onset x 15	0.375
	Onset x 30	0.029*
	Onset x 180	0.001*
	15 × 30	0.999
	15 × 180	0.999
	30 × 180	0.999
Without disease	Onset x 15	0.001*
	Onset x 30	0.001*
	Onset x 180	0.001*
	15 × 30	0.999
	15 × 180	0.999
	30 × 180	0.999

* p ≤ 0,05

For the group “without associated disease”, we recorded a change in the mean values obtained with 15, 30 and 180 days (which are equal) in relation to the initial value.

## DISCUSSION

When describing two cases of sudden idiopathic sensorineural hearing loss in patients with diabetes, Jorgensen et al.^9^ suggested that the vascular alterations which happened as complications of diabetes would also involve inner ear vessels, which would explain the etiology of sudden idiopathic sensorineural hearing loss in diabetic patients. Nonetheless, so far, the role of diabetes mellitus, systemic arterial hypertension and dyslipidemias in cases of sudden idiopathic sensorineural hearing loss remain unexplained^1^,^10^. The role of these disorders in the evolution and prognosis of patients with sudden idiopathic sensorineural hearing loss was the motivation we had for this study.

In our study, the sample size was reduced because of our strict inclusion and exclusion criteria. Nevertheless, it was the only way to assess the evolution of patients with sudden idiopathic hearing loss in the presence of systemic arterial hypertension, diabetes mellitus and/or dyslipidemias throughout time, and not only to assess the hearing improvement with the treatment adopted.

In our studies there was no statistically significant difference concerning sudden idiopathic sensorineural hearing loss between men and women, according to the literature^2^. Initially, the men and women of the sample were analyzed separately, however, since there were no statistical differences between the results, the patients were grouped and assessed together.

In our study we assessed the presence of symptoms such as tinnitus, dizziness and ear fullness. The prevalence of tinnitus was 83%, dizziness was 51% and ear fullness was present in 20% of our patients^1^,^11^. In the isolate assessment of the groups with and without associated disease, regarding the presence of these symptoms, there were no statistically significant differences between the groups and without influence in the auditory recovery with the presence of tinnitus, dizziness and ear fullness. Our findings show the prevalence of these symptoms, however without worsening in hearing prognosis in both groups.

In the patients “with associated disease”, systemic arterial hypertension was the most prevalent, followed by Dyslipidemia and diabetes mellitus. In three patients there was an association with systemic arterial hypertension and diabetes mellitus. In the study by Duck et al.^12^, evaluating the association of arterial hypertension with diabetes mellitus and in the studies by Morizono and Paparella^13^ and by Yan-Lin and Yuan-Ping^14^, evaluating the association of arterial hypertension with dyslipidemias, the association of these systemic diseases had an addictive effect on the worsening of the hearing loss. In our three patients, when evaluated alone, it was not possible to confirm this effect.

In a more detailed evaluation we found that of the four patients with alterations in the fundus of the eye exams in the group of associated diseases, three had association with hypertension and diabetes and the other patient had only diabetes. Jorgensen and Buch^15^ found hearing loss, twice more common in diabetic patients with retinopathy. Axelsson et al.^16^ suggested the association of eye microangiopathies which happened in diabetes with different types of hearing loss, and Kurien et al.^17^ also observed that the lack of diabetes control, assessed through eye complications, were important in the evolution of the hearing loss. Assessing again each one of the patients alone it was not possible to identify the relationship between ocular alterations of the eye fundus and the worst auditory evolution of the patients. Nonetheless, dealing specifically with sudden idiopathic sensorineural hearing loss the findings from Wilson et al.^18^ confirmed our results since they did not find such association as well.

As we continued to assess the patients, we found that the age of the group with associated disease is statistically higher in the group without associated disease. In their study, Axelsson et al.^19^ had already described a worsening in the hearing loss with aging without, however, finding an association with diabetes control and duration. Brohem et al.^20^ studied hypertensive patients complaining of hearing usually associated to their more advanced age. Marchiori et al.^21^ considered systemic arterial hypertension and advanced age as isolated risk factors for sensorineural hearing loss. Concerning sudden idiopathic sensorineural hearing loss we could not isolate the most advanced age as an isolated risk, since the most advanced age coexisted in patients with associated diseases.

In the hearing improvement evolution in patients with sudden idiopathic sensorineural hearing loss, the findings from Yamamoto et al.^22^ and Penido et al.^1^, were assessed through the hearing improvement rate and its results, with an auditory recovery above 50%, which were 71.9% and 67.5% respectively. In our two groups, the mean values of the improvement rate are around 40% and without statistical difference between the groups, although we noticed a trend towards a better evolution for the group without associated disease. Both values contradict literature results; nonetheless, we must consider that for the group with associated disease and the group without associated disease the mean time of treatment onset was of 11 and 15 days, respectively. Such values justify the worsening in the improvement rates, since in the two reported studies^1^,^22^the best results were obtained with the treatment onset in up to seven days after the onset of sudden idiopathic sensorineural hearing loss symptoms.

Still in the auditory improvement evolution, Barros and tal.^12^ concluded that the speech recognition index could provide data as to sudden idiopathic hearing loss. This is confirmed in the evaluation of our patients, when the speech recognition index in both groups presented a statistically significant difference regarding evolution and in the interaction between the different assessment periods. In the group of associated disease, the improvement in the speech recognition index was more relevant on the first 30 days after treatment onset while in the group without associated disease only the first fifteen days were the most relevant. This data shows a slower improvement in the group of patients with associated disease.

In following the patients with sudden hearing loss along time, Furuhashi et al.^8^ studied 1798 patients who had sudden sensorineural hearing loss between the years of 1972 and 2000, and found only 4 cases of ipsilateral recurrence (0.22%) and 10 of contralateral involvement (0.55%), with a time between the episodes varying between nine months and 14 years. In our study we found recurrence in the group with associated disease, nevertheless there were two cases of ipsilateral recurrence (10% of the group and 5.7% of the sample) with more than six months and one year of time between the episodes. This data suggests the importance of the associated diseases in the evolution and prognosis of sudden idiopathic sensorineural hearing loss.

The inner ear evaluation of the patients could provide more data than interfere in the evolution of our patients. In autopsies of diabetic patients, Makishima and Tanaka^23^ carried out a histopathology study of the temporal bones and found alterations, especially in the peripheral nerves, characterized by fibrous thickening and narrowing of the stria vascularis vessel lumen. The same histological findings were found by Smith et al.^24^ studying diabetic rats. Bachor et al.^25^, also doing histological studies of temporal bones, concluded that, in some cases the inner ear vascular variation can be associated with hearing alterations. Nonetheless, the histopathology exam in our study was not possible to be carried out because it was not possible to collect in vivo material. Thus, the evaluation of the labyrinthine alterations and that of the central nervous system was based on MRI.

In the assessment of patients with sudden idiopathic sensorineural hearing loss, Ramos et al.26 found alterations in the MRI of 46.9% of the patients, and the subcortical and hypertensive periventricular lesions – matching those of cerebral microangiopathy, represented 68.4% of the central alterations affecting patients which mean age was 65.2 years. In a histopathological study with hypertensive rats, Junker et al.^27^ also found cerebral microangiopathy in regions already affected by hypertension. In our study, MRI also showed the presence of cerebral microangiopathy in 13 patients (37.14%) of the sample and proved to be more prevalent in the group with associated disease (85% of the total), with a statistical difference. The two patients from the group without associated diseases (15%) with microangiopathy had ages above 65 years, which did not necessarily happen in the group with associated disease. This data shows the role age and associated diseases play in cerebral microangiopathy in the patients with sudden idiopathic sensorineural hearing loss.

## CONCLUSION

Sudden idiopathic sensorineural hearing loss in the presence of systemic arterial hypertension, diabetes mellitus and dyslipidemias, in older individual, is associated to a higher prevalence of cerebral microangiopathy in the MRI and it is implied in an auditory recovery with a slower evolution in the speech recognition index.

## References

[1] Penido N.O., Ramos H.V.L., Barros F.A., Cruz O.L.M., Toledo R.N. (2006). Fatores clínicos, etiológicos e evolutivos da audição na surdez súbita. Braz J Otorhinolaryngol..

[2] Lazarini P.R., Camargo A.C.K. (2006). Surdez súbita idiopática:aspectos etiológicos e fisiopatogênicos. Braz J Otorhinolaryngol..

[3] Schuknecht H.F., Donovan E.D. (1986). The pathology of idiopathic sudden sensorial hearig loss. Arch Otolaryngol..

[4] Maassab HF. (1973). The role of viruses in sudden deafness. Adv *Otorhinolaryngol..

[5] Pitkäranta A., Julkunen I. (1998). Suddendeafness: Lack evidence for systemic viral infection. Otolaryngol Head Neck Surg..

[7] Wei B.P.C., Mubiru S., *O'Leary S. (2006). Steroids for idiopathic sudden sensorineural hearing loss. The Cochrane Library.

[8] Yamamoto M., Kanzaki J., Ogawa K., Ogawa S., Tsuchihashi N. (1994). Evaluation of hearing recovery in patients with sudden deafness. Acta Otolaryngol..

[9] Jorgensen M.B. (1959). Sudden Loss of inner ear function in the course of long-standing diabetes mellitus. Acta Otolaryngol..

[10] Cullen J.R., Cinnamond M.J. (1993). Hearing loss in diabetes. J Laryngol Otol..

[11] Barros F.A., Penido N.O., Ramos H.V.L., Sanchez M.L., Fukuda Y. (2003). Audiological evaluation of twenty patients receiving pentoxifilline and prednisone after sudden deafness: prospective study. Int Tinnitus J..

[12] Duck S.W., Prazma J., Bennett P.S., Pillsbury H.C. (1997). Interaction between hypertension and diabetes melito in the pathogenesis of sensorineural hearing loss. Laryngoscope..

[13] Morizono T., Paparella M.M. (1978). Hypercholestoremia and auditory dysfunction. Ann Otol Rhinol Laryngol..

[14] Lin C.Y., Ping D.Y. (1999). Relationship between hypertension and hearing disorders in the elderly. East Afr Med J..

[15] Jorgensen M.B., Buch N.H. (1962). Studies on inner-ear function and cranial nerves in diabetics. Acta Otolaryngol..

[16] Axelsson A., Sigroth K., Vertes D. (1978). Hearing in diabetics. Acta Otolaryngol..

[17] Kurien M., Thomas K., Bhanu T.S. (1989). Hearing threshold in patients with diabetes melito. J Laryngol Otol..

[18] Wilson W.R., Laird N J.S., Young G.M., Kavesh D.A., Macmeel J.W. (1982). The relationship of idiopathic sudden hearing loss to diabetes melito. Laryngoscope..

[19] Axelsson A., Fargerberg S.E. (1968). Auditory function in diabetics. Acta Otolaryngol..

[20] Brohem V.M.A., Caovilla H.H., Ganança M.M. (1996). Dos sintomas e achados audiológicos e vestibulares em indivíduos com hipertensão arterial. Acta AWHO..

[21] Marchiori L.L.M., Filho E.A.R., Matsuo T. (2006). Hipertensão como fator associado à perda auditiva. Braz J Otorhinolaryngol..

[22] Yamamoto M., Kanzaki J., Ogawa K., Ogawa S., Tsuchihashi N. (1994). Evaluation of hearing recovery in patients with sudden deafness. Acta Otolaryngol..

[23] Makishima K, Tanaka K. Pathological changes of the inner ear and central auditory path way in diabetics. Presented at the 12thAnnual Congress of the Japan Audiological Society. 1967:218–28.

[24] Smith T.L., Raynor E., Prazma J., Buenting J.E., Pillsbury H.C. (1995). Insulin-dependent diabetic microangiopathy in the inner ear. Laryngoscope..

[25] Bachor E., Selig Y.K., Jahnke K., Rettinger G., Karmody C. (2001). Vascular variations of the inner ear. Acta Otolaryngol..

[27] Junker U., Jaggi C., Bestetti G., Rossi G.L. (1985). Basement membrane of hypothyalamus and cortex capillaries from normotensive and spontaneously hypertensive rats with streptozotocin- induced diabetes. Acta Neuropathol..

